# An index of non-sampling error in area frame sampling based on remote sensing data

**DOI:** 10.7717/peerj.5824

**Published:** 2018-11-12

**Authors:** Mingquan Wu, Dailiang Peng, Yuchu Qin, Zheng Niu, Chenghai Yang, Wang Li, Pengyu Hao, Chunyang Zhang

**Affiliations:** 1 The State Key Laboratory of Remote Sensing Science, Institute of Remote Sensing and Digital Earth, Chinese Academy of Sciences, Beijing, China; 2 Laboratory of Digital Earth Sciences, Institute of Remote Sensing and Digital Earth, Chinese Academy of Sciences, Beijing, China; 3 Aerial Application Technology Research Unit, USDA-Agricultural Research Service, College Station, TX, United States of America; 4 Key Laboratory of Agricultural Remote Sensing, Ministry of Agriculture, China/Institute of Agricultural Resources and Regional Planning, Chinese Academy of Agricultural Sciences, Beiijng, China; 5 National Engineering Center for Geoinformatics, Institute of Remote Sensing and Digital Earth, Chinese Academy of Sciences, Beijing, China

**Keywords:** Crops, Landsat, Non-sampling errors, Remote sensing, Crop area statistics

## Abstract

Agricultural areas are often surveyed using area frame sampling. Using non-updated area sampling frame causes significant non-sampling errors when land cover and usage changes between updates. To address this problem, a novel method is proposed to estimate non-sampling errors in crop area statistics. Three parameters used in stratified sampling that are affected by land use changes were monitored using satellite remote sensing imagery: (1) the total number of sampling units; (2) the number of sampling units in each stratum; and (3) the mean value of selected sampling units in each stratum. A new index, called the * non-sampling error by land use change index* (NELUCI), was defined to estimate non-sampling errors. Using this method, the sizes of cropping areas in Bole, Xinjiang, China, were estimated with a coefficient of variation of 0.0237 and NELUCI of 0.0379. These are 0.0474 and 0.0994 lower, respectively, than errors calculated by traditional methods based on non-updated area sampling frame and selected sampling units.

## Introduction

In many countries, accurate and timely national-level agricultural statistics are provided by agricultural statistics services such as the US Department of Agriculture’s National Agricultural Statistics Service (NASS), the Italian National Statistical Institute (Istat), the Agrifood and Fishery Information Service of Mexico (SIAP), the National Bureau of Statistics of China (NBS), and the Iranian Ministry of Agrculture’s Agricultural Statistics Information Division (ASID; Allen, 1990; Alonso, Soria & Gozalo, 1991; Gallego, 1999; Kussul et al., 2012; Pradhan, 2001; Wu & Li, 2004). The traditional method of producing agricultural statistics (Benedetti et al., 2010; Tsiligirides, 1998) is, firstly, to create a census list based on registers or other kinds of administrative data. The census list is then used to create a crop survey regime. The list of sampling units is usually not updated for around 5–10 years. Data from selected sampling units are collected by face-to-face interviews, telephone interviews, or emails. Overall, this method is very costly and, therefore, difficult for developing countries to use. In developing countries, agricultural statistics are often produced by aggregating administrative data, which results in low data quality and quantity (World Bank, FAO & United Nations Statistical Commission, 2011; FAO, World Bank & United Nations Statistical Commission, 2012).

To overcome these problems, new methods have been developed based on remote sensing (RS), geographic information systems (GIS) and global positioning systems (GPS). Since the early 1970s, with funding from the National Aeronautics and Space Administration (NASA), the National Association of Secretaries of State (NASS) has studied methods for collecting agricultural statistics based on Landsat data (Alonso, Soria & Gozalo, 1991; Bellow & Graham, 1992; Gonzalez-Alonso et al., 1997). In Europe in the late 1980s, the Monitoring Agriculture with Remote Sensing (MARS) project was carried out to develop tools for large-scale remote sensing operational applications, such as the Italian AGRIT Project (Alonso, Soria & Gozalo, 1991; Gallego, 1999; Kussul et al., 2012). In China, remote sensing has been used to map crops since the late 1970s. It has also been used in agricultural studies since the late 1990s and in operational applications for the NBS since 2009 (Liu et al., 2015; Wu & Li, 2004; Wu & Li, 2012; Wu et al., 2014).

Remote sensing has two main uses in agricultural data collection from an area sampling frame that is a set of land elements, which may be either points or segments of land. Firstly, at the design level, remote sensing can be used to define sampling units and strata (Beuchle et al., 2015; Boryan et al., 2011; Carfagna & Gallego, 2005; Kim et al., 2018; Wu & Li, 2004). Secondly, classified remote sensing data can also be a useful source of auxiliary data for field ground surveys and area estimation (Husak et al., 2008; Liu et al., 2015; Vintrou et al., 2012; Wu et al., 2014). For example, in China, satellite remote sensing images or aerial images with spatial resolutions greater than 2.5 m are widely used to carry out a complete census or area sampling frame (Liu et al., 2015; Wu et al., 2014). These high spatial resolution remote sensing images are also very useful in field surveys of sampled units. Medium spatial resolution remote sensing classification data, mapped using Landsat and Gaofen No.1 (GF-1) satellite data, is also widely used to extract parameters for stratification (Liu et al., 2015; Wu et al., 2014; Yang et al., 2007). In Europe, aerial photographs and high-precision satellite images are used for the identification of all agricultural parcels in the Integrated Administration and Control System (IACS), which is used for the management and control of payments to farmers by member states according to the Common Agricultural Policy (https://ec.europa.eu/agriculture/direct-support/iacs_en).

Although remote sensing has many advantages in terms of agricultural statistics, it also has disadvantages. Firstly, in order to build a complete area sampling frame, satellite images with spatial resolutions greater than 2 m are required. However, such images are expensive, making it very costly to compile a complete area frame for national applications (Gallego et al., 2014). Furthermore, the workload required is very large, especially since high-accuracy area sampling frame can only be built using manual visual interpretation methods (Shuang & Jinshui, 2013). Thus, overall, a complete area sampling frame based on high spatial resolution remote sensing data is very costly in both money and time (Boryan et al., 2011). Most important, however, is the fact that this data is used for long time periods (5–10 years), such that changes in land cover and usage can cause significant non-sampling errors derived from under- or over-coverage (Benedetti et al., 2010). Non-sampling errors are defined as errors that may arise over the complete survey process (i.e., from frame development to data analysis). They may be systematic or random, but are unrelated to random sampling errors such as sample frame over- or under-coverage, and errors resulting from poorly-worded questionnaires (FAO, 2015). Thus, there is a clear need for a method that can estimate non-sampling errors reliably, particularly to evaluate whether non-updated area sampling frame can still be used.

Finally, since remote sensing satellites can periodically image the earth, they are very suitable for monitoring land cover and usage changes (Beuchle et al., 2015; Richards, Gallego & Achard, 2000; Stern, Doraiswamy & Raymond Hunt, 2012; Vintrou et al., 2012; Vittek et al., 2014). To solve this problem, we propose a method that uses remote sensing to evaluate the usability of non-updated area sampling frame and to estimate non-sampling errors. The overall objectives of this study are to: (1) propose a method that uses remote sensing to evaluate the usability of non-updated area sampling frame; (2) analyze the influences of these non-updated sampling units on area frame sampling; and (3) propose a land use change index (NELUCI) that describes non-sampling errors.

## Study Area and Data Processing

### Study area

Xinjiang is China’s major cotton growing region and produces nearly 60% of China’s cotton. From 2009, the cotton area was subject to area frame sampling via remote sensing by the Chinese Bureau of Statistics.

**Figure 1:**
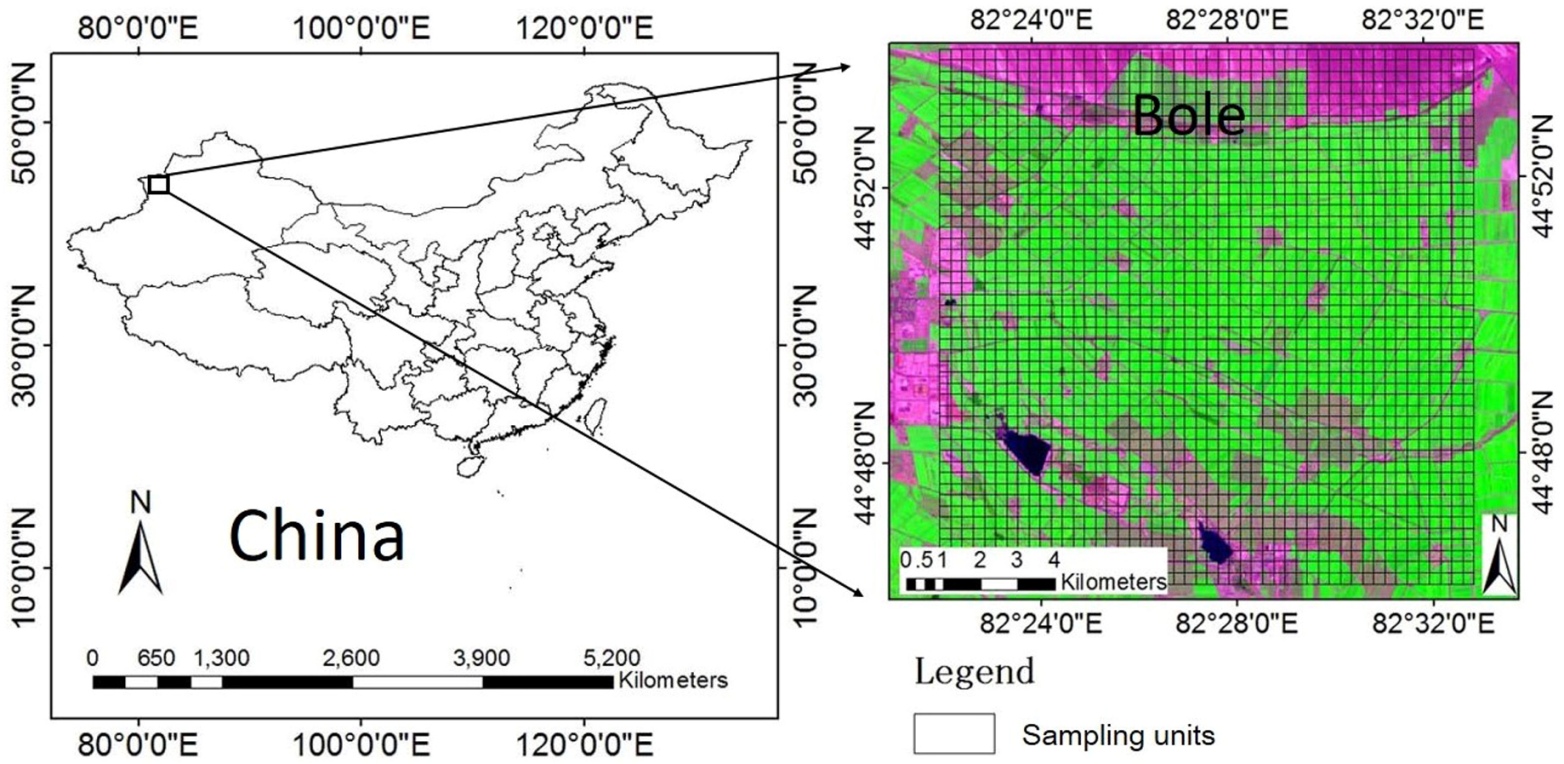
Study area location with Landsat Operational Land Imager (OLI) image (SWIR 2, NIR and red bands) acquired on July 22, 2015, provided by the USGS. The sampling units were set to a 300 m × 300 m grid based on a 300 m × 300 m fishnet created in ARCGIS 10.

A 14.4 km × 14.4 km square area located east of Bole City, Xinjiang Province, China, was selected as the study area (Fig. 1). The latitude and longitude of this area ranged from 44°44′N to 44°53′N, and 82°19′E to 82°34′E. The main land-use types within this area are farmland, desert, forest, residential, and water bodies. Most farmland is planted with cotton, with some corn and spring wheat crops. All of crops are sown in April. Spring wheat is harvested in July, while corn is harvested in September and cotton is harvested in October. Data on cotton in this area was collected using area frame sampling methods from 2009.

### Image data and pre-processing

This study used four Landsat-Thematic Mapper (TM) images (2011), four Landsat-Operational Land Imager (OLI) images (2015; provided by the US Geological Survey; USGS), and one 2 m image captured on a GF-1 panchromatic multispectral camera (PMS; acquired on May 7, 2015; Table 1). All of the images covering this study area were clear of cloud cover. Both the Landsat-TM and Landsat-OLI images were L1T productions. The GF-1 PMS imagery was in Level 1A. All of the images were atmospherically corrected using the Fast Line-of-Sight Atmospheric Analysis of Spectral Hypercubes (FLAASH) atmospheric correction model in ENVI5.3. All the atmospherically-corrected images were georeferenced using a second-order polynomial warping approach with 40 ground control points (GCPs) selected from a 1:10,000 topographic map. The positional errors of all images were lower than 0.7 Landsat pixels or 0.6 GF-1 PMS pixels. Thereafter, normalized difference vegetation indices (NDVIs) were calculated and layered into two multi-temporal Landsat NDVI datasets according to their respective years. Finally, crops in the study area in 2011 and 2015 were classified from the multi-temporal Landsat NDVI data using the maximum likelihood method with data from 40 plots measured by field surveys as areas of interest (AOIs). Furthermore, crops in 2015 also were classified from multispectral GF-1 PMS imagery using the maximum likelihood method with the same AOIs.

**Table 1:** Landsat images used in this study.

	**Landsat-TM**	**Landsat-OLI**	**GF-1 PMS**
Acquisition date	05/24/2011	06/20/2015	05/07/2015
	07/11/2011	07/22/2015	
	07/27/2011	09/08/2015	
	09/13/2011	09/24/2015	
Path/row	146/29	146/29	62/79

**Figure 2:**
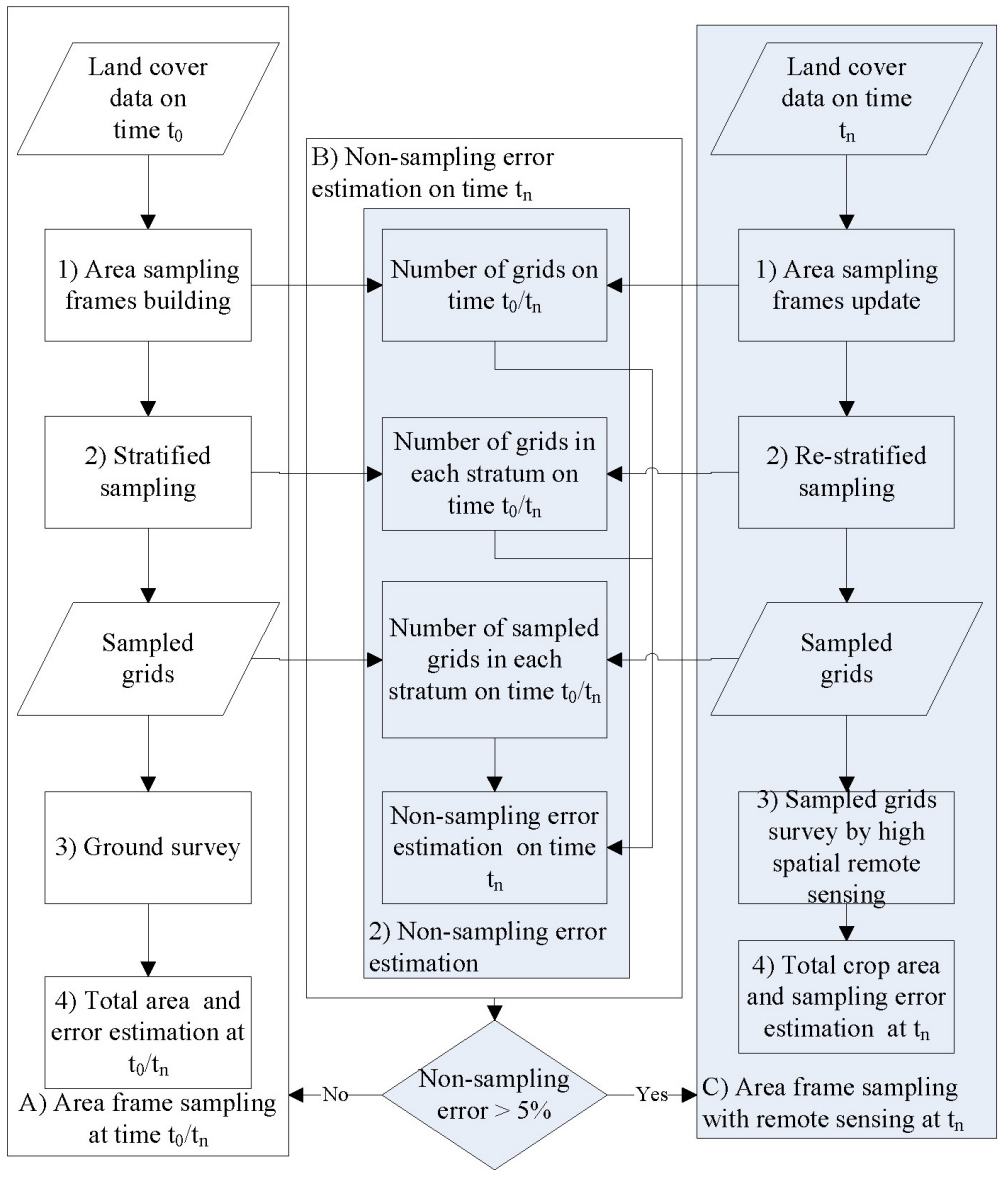
Flow chart of (A) conditional area frame sampling at time *t*_0_∕*t*_*n*_; (B) non-samplingerror estimate at time *t*_*n*_; and (C) area frame sampling with remotesensing at time *t*_*n*_.

## Methods

In general, there are four steps for conditional area frame sampling using remote sensing data (Fig. 2A). Firstly, a complete area sampling frame is built according to two steps (Vintrou et al., 2012). (1) The land cover types (cotton or not cotton) of each plot were identified using multispectral Landsat data from 2011 using the spectral angle mapper (SAM) classification method; (2) square segments were created by overlapping a regular grid with the land cover data obtained in steps one (Gallego et al., 2014). Secondly, a stratified sampling method was used to sample segments or grids, using the cotton area ratio as a hierarchical basis (Gonzalez-Alonso et al., 1997). Thirdly, cotton areas within each sampled area frame were assessed by ground surveys. Finally, the total crop area of the census area and the sampling errors were estimated. To reduce costs, this complete area sampling frame will be used for the next five to ten years (named conditional area frame sampling at time *t*_*n*_; Boryan et al., 2011). This means that only the third and fourth steps need to be completed over the subsequent five to ten years. Although it would reduce costs, not updating the area sampling frame over time causes numerous problems, such as: (1) the area sampling frame may be under- or over-covered at time *t*_*n*_; (2) the sampled grids at time *t*_0_ may not be suitable for area frame sampling at time *t*_*n*_; and (3) there may be significant non-sampling errors. Therefore, there is a need to estimate the non-sampling errors of conditional area frame sampled at time *t*_*n*_ to determine whether their results can be used. Obviously, if the non-sampling errors are high (for example, >5%, which is the minimum accuracy required by the Chinese Bureau of Statistics), area frame re-sampling or calibration is needed at time *t*_*n*_.

In order to evaluate the usability of conditional area sampling frame data and estimate non-sampling errors caused by changes in land cover and usage types, we developed a novel method based on remote sensing technology. This method involves two steps (Figs. 2B and 2C): (1) area frame sampling based on remote sensing data at time *t*_*n*_; and (2) non-sampling error calculations.

The inputs to this method include two sets of medium-resolution land cover or land use data, one set of high spatial resolution data, and two sets of field survey data comprised of old sampled grids. Previous land cover/use data and field survey data were used for area frame sampling at time *t*_0_. Later land cover/use data and field survey data were used for area frame sampling of the year for which predictions were needed, which was usually the current year.

### Area frame sampling with remote sensing data

Area frame sampling with remote sensing data was conducted in four steps: (1) area sampling frame update; (2) re-stratified sampling; (3) sampled grids survey by high spatial resolution remote sensing data with ground survey data; and (4) total crop area and sampling error calculation.

There are several differences between area frame sampling with remote sensing data at time *t*_*n*_ and conditional area frame sampling at time *t*_*n*_. Firstly, area frame sampling with remote sensing data at time *t*_*n*_ is based on land cover data at time *t*_*n*_. As the area sampling frame is updated to time *t*_*n*_, it is more complete than older area sampling frame and will, therefore, has lower non-sampling errors. Secondly, stratified sampling is also redone based on the updated area sampling frame to sample new grids for each stratum. Thus, the area sampling frame and sampled grids are different to those used in conditional methods. Thirdly, conditionally, the cotton areas of sampled grids were determined by ground survey. However, the cotton area of newly sampled grids cannot be determined using this method because the newly sampled grids were different to the old ones. Thus, the cotton area of each newly sampled grid was determined from a land cover map classified by 2 m GF-1 PMS data using ground survey data collected at time *t*_*n*_ in the location of the older sampled grids as AOIs.

#### Updating area sampling frame

The sampling units of an area frame are portions of territory, usually termed *segments*, that are defined as area units with regular geometric shapes or are delimited by physical boundaries (Carfagna & Gallego, 2005). Both geometric shapes and physical boundaries can be easily extracted from remote sensing images. Thus, a complete area sampling frame can always be built using remote sensing images. In this paper, the area sampling frame was a set of grids within cotton cropping areas.

Before evaluating the usability of the non-updated area sampling frame in the 2015 crop area statistics, a complete area sampling frame was built using the 2011 cotton area map (Liu et al., 2015; Wu et al., 2014). This was carried out in three steps. Firstly, Moran’s I index, which is a measure of spatial autocorrelation, was calculated using the spatial autocorrelation tools in ARCGIS 10.1, with the 2011 cotton area map used as input (Wu et al., 2014). The results showed that the spatial resolution with the lowest spatial autocorrelation was 300 m. Thus, secondly, the sampling units were set as a 300 m × 300 m grid. Then, a 300 m × 300 m fishnet was created using the fishnet tools in ARCGIS 10.1, using the 2011 cotton area map as input. Finally, a complete 300 m × 300 m area sampling frame were built by removing all the grid units that did not contain cotton. The value of each grid was set to the cotton area in the grid.

To update this area sampling frame, a new 300 m × 300 m fishnet was made and overlaid on the 2015 cotton area map. A new complete 300 m × 300 m area sampling frame was built by removing all the grid units without cotton in 2015. The values of each grid were also updated according to the 2015 data.

#### Re-stratified sampling

For 2011, all the 300 m × 300 m grids were categorized into five strata according to the cotton area ratio in each grid unit. Then, 24 grid units were selected using a stratified sampling method with 95% confidence and 5% relative error conditions.

Compared with the 2011 data, the 2015 land cover/use data changed, thus leading to changes in the area sampling frame. This change had three consequences for the area sampling frame. Firstly, it led to changes in the total number of grids. Secondly, it changed the number of grids in each stratum. Thirdly, the values of some grids that were sampled in order to represent a stratum, were not within the range of values expected for that stratum. Consequently, some of the grids may have been assigned to a stratum other than the one they were supposed to represent. Importantly, if incomplete and non-updated area sampling frames, and non-updated sampled grids are still used, non-sampling errors will almost certainly be introduced. Therefore, there is a specific need to re-stratify.

Remote sensing is a very useful tool for monitoring land cover or land use changes and, thus, is also suitable for monitoring area sampling frame changes (Duveiller & Defourny, 2010; Fritz et al., 2015; Stern, Doraiswamy & Raymond Hunt, 2012). For example, changes to area sampling frames are easy to monitor due to remote sensing observations of variations in cropping areas.

Due to land cover or land use changes, the total number of grids and the number of sampled grids in each stratum may change. Therefore, re-stratified sampling was conducted based on the new area sampling frame to sample new grids in each stratum. Obviously, the new sampled grids in each stratum will differ from the older ones. Moreover, owing to land cover changes, the number of grids may also be different than in conditional methods. Hence, the number of sampled grids in each stratum also may be different than that resulting from conditional methods.

#### Sampled grids based on high spatial resolution remote sensing and ground survey data

After the sampling units were selected, a ground survey of the cotton areas in a previously sampled grids was conducted for years 2011 and 2015. The plots in each sampled grid were segmented by an expert remote sensing image interpreter using high spatial resolution data. Then, the features of all plots were surveyed on the ground using a GPS receiver. There were 60 plots surveyed in 2011 and 2015. The actual proportion of cotton areas in each sampled grids were then calculated.

Since the new sampled grids were different than the old ones, a new method of determining the values of the new grids was applied using GF-1 remote sensing data. This new method was conducted over three steps. Firstly, cotton area data based on 2015 ground surveys were used to identify AOIs. Then, the cotton areas encompassed by the study area were mapped using a maximum likelihood method with total accuracy of 92.3%. Finally, the cotton area in each sampled grid was counted by overlaying a cotton map, and the corresponding values were set to each frame.

#### Total crop area and sampling error calculation

When determining the values of new sampled grids from high spatial resolution remote sensing images, the total cotton area, $\hat {Y}$, can be calculated according to the stratified sampling method:

(1) \begin{eqnarray*}& & \hat {Y}=\sum _{h=1}^{L}{N}_{h}{\overline{y}}_{h},\end{eqnarray*}

where ${\overline{y}}_{h}$ is the mean cotton area of sampled frames in the *h*th stratum; *L* is the number of strata; and *N*_*h*_ is the total number of area sampling frames within the *h*th stratum. The variance, $v(\hat {Y})$, and the coefficient of variation, *CV*, are calculated as follows (Carfagna & Arti, 2007):

(2) \begin{eqnarray*}& & v(\hat {Y})=\sum _{h=1}^{L}{N}_{h}^{2} \frac{1-{f}_{h}}{{n}_{h}} {S}_{h}^{2},\end{eqnarray*}

(3) \begin{eqnarray*}& & {f}_{h}= \frac{{n}_{h}}{{N}_{h}} ,\end{eqnarray*}

(4) \begin{eqnarray*}& & {S}_{h}^{2}= \frac{1}{{n}_{h}-1} \sum _{i=1}^{{n}_{h}}({y}_{hi}-{\overline{y}}_{h})^{2},\end{eqnarray*}

(5) \begin{eqnarray*}& & CV=\sqrt{v(\hat {Y})}/\hat {Y},\end{eqnarray*}

where *f*_*h*_ is the sampling proportion of the *h*th stratum; *n*_*h*_ is the number of sampled grids in the *h*th stratum; *y*_*hi*_ is the value of the *i*th sampled grid of the *h*th stratum; ${\overline{y}}_{h}$ is the mean value of the sampled grids in the *h*th stratum; and ${S}_{h}^{2}$ is the variance of the sampled grids in the *h*th stratum.

### Non-sampling error calculation

The CV is a good parameter with which to measure the sampling error. However, the non-sampling error, caused by sampling new grids from non-updated area frames, does not influence the CV. Therefore, a new parameter is needed that can indicate the extent of non-sampling error. According to Eq. (1), the total crop area $\hat {Y}$ can be estimated by the mean value of the sampled grids and by the number of grids within each stratum. Both parameters are influenced by the non-updated area sampling frame and also by the non-updated sampled grids. Due to land coverage or land use changes, both the total number of grids and the number of grids in each stratum may change. This will lead to a change in the parameter *N*_*h*_. Alongside this, the values of sampled grids also may change due to land coverage/use changes. Indeed, large changes can even lead to the values of some sampled grids falling into the range of those in other strata. This means that the sampled grids that ostensibly represent the *i*th stratum actually do not. If the values of these unrepresentative sampled grids are still used to represent the *i*th stratum, the mean value of the *i*th stratum will change substantially, resulting in the introduction of large non-sampling errors. We propose a new parameter to evaluate non-sampling errors, which can be easily extracted from remote sensing images. According to Eq. (1), the non-sampling error from the land use change index (NELUCI) is defined as follows:

(6) \begin{eqnarray*}& & \mathrm{NELUCI}=E\text{_}{\hat {Y}}_{i,j}/{\hat {Y}}_{j}\times 100\text{%},\end{eqnarray*}

(7) \begin{eqnarray*}& & E\text{_}{\hat {Y}}_{i,j}=\sum _{h=1}^{L}abs \left( {N}_{j,h}{\overline{y}}_{j,h}-{N}_{i,h}{\overline{y}}_{i,h} \right) ,\end{eqnarray*}

where *i* and *j* are the sampling times. Thus, $E\text{_}{\hat {Y}}_{i,j}$ represents the error arising from changes in land use. From land use data mapped by remote sensing images at times *i* and *j*, and using the method mentioned in ‘Sampled Grids based on High Spatial Resolution Remote Sensing and Ground Survey Data’, the values of *N*_*i*,*h*_ and *N*_*j*,*h*_ for each sampling unit can be easily calculated by overlaying land use data at times *i* and *j* on the area sampling frame. Thereafter, the values of ${\overline{y}}_{i,h}$ and ${\overline{y}}_{j,h}$ can be calculated using the values of sampled grids at times *i* and *j*.

## Results

### Results of conditional area frame sampling method

Through ground survey, the values of each sampled grid in 2015 were obtained. Then, the mean and variance of each stratum were calculated. Finally, the total cotton area in the study area and associated errors were estimated (Table 2). Table 2 shows that the CV and NELUCI were both higher than 5% due to the sampled grids not being updated. Eight sampled grids lost their representativeness due to land use changes from 2011 to 2015.

**Table 2:** Results of area sampling with non-updated area sampling frame. Five strata were defined according to the cotton-to-non-cotton area ratio in each grid unit.

**Stratum number**	**Total no. of area frames (*N*_*h*_)**	**Weight (*f*_*h*_)**	**Variance of each stratum (${S}_{h}^{2}$)**	**No. of sampled area frames (*n*_*h*_)**	**Mean value of sampled area frames**	**Variance of sampled area frames (${s}_{h}^{2}$)**	$ \left( 1-{f}_{h} \right) /{n}_{h}$	$ \left( \left( 1-{f}_{h} \right) /{n}_{h} \right) \times {s}_{h}^{2}\times {N}_{h}^{2}$	**Total crop area** ($\hat {Y}$) (Sampling Units, 0.09 km^2^)	**CV**	**NELUCI**
1	772	0.3351	0.0023	8	0.1650	0.1092	0.1237	8050.4401	1337.5893	0.0711	0.1373
2	207	0.0898	0.0032	2	0.4500	0.0242	0.4948	513.0721			
3	266	0.1155	0.0033	3	0.7700	0.0016	0.3299	37.3434			
4	496	0.2153	0.0031	5	0.7760	0.0020	0.1979	96.4075			
5	563	0.2444	0.0047	6	0.9367	0.0064	0.1649	335.9725			

### Results of the proposed method

By the process of overlaying area sampling frame with 2015 land cover maps, *N*_*h*_ and the variance of each stratum were calculated. With the area sampling frame updated and the sampled grids re-sampled and surveyed by 2 m high spatial resolution remote sensing imagery with AOIs based on ground survey data, the total cotton area in the study area and associated errors were successfully estimated (Table 3). Based on this proposed method, the total cotton area was estimated to be 109.86 km^2^ (1220.62 grids) with a CV of 0.0237 and a NELUCI of 0.0379, both of which are lower than 5%. Usually, the CV is used to evaluate sampling errors, with a lower CV representing higher accuracy. By comparing Tables 2 and 3, it can be found that without updating the sampled grids, a high CV was associated with the conditional area frame sampling method. It also can be seen from Tables 2 and 3 that the accuracy evaluations by CV and NELUCI are consistent with each other. They are both higher than 5% in the conditional method, and both lower than 5% in the proposed method. However, the NELUCI is larger than the CV in both methods. This is because the NELUCI not only considers the sampling errors, but is also influenced by non-sampling errors. Thus, the proposed method is effective for estimating the total cotton area, and produces lower sampling and non-sampling errors.

**Table 3:** Results of area sampling with updated area sampling frame. Five strata were defined according to the cotton-to-non-cotton area ratio in each grid unit.

**Stratum no.**	**Total no. of area frames (*N*_*h*_)**	**Weight (*f*_*h*_)**	**Variance of each stratum (${S}_{h}^{2}$)**	**No. of sampled area frames (*n*_*h*_)**	**Mean value of sampled area frames**	**Variance of sampled area frames (${s}_{h}^{2}$)**	$ \left( 1-{f}_{h} \right) /{n}_{h}$	$ \left( \left( 1-{f}_{h} \right) /{n}_{h} \right) \times {s}_{h}^{2}\times {N}_{h}^{2}$	**Total crop area** ($\hat {Y}$) (Sampling Units, 0.09 km^2^)	**CV**	**NELUCI**
1	670	0.2908	0.0023	6	0.0183	0.0020	0.1649	149.3086	1220.6200	0.0237	0.0379
2	183	0.0794	0.0032	2	0.3000	0.0032	0.4948	53.0242			
3	270	0.1172	0.0033	1	0.5600	0.0033	0.9896	236.7360			
4	488	0.2118	0.0031	6	0.7583	0.0011	0.1649	41.5029			
5	693	0.3008	0.0047	9	0.9122	0.0068	0.1100	358.7817			

## Discussion

### Analysis of whether to update area sampling frames

When using non-updated area sampling frame for agricultural statistics, three of their parameters will be affected: (1) total number; (2) number in each stratum, and (3) the mean value in each stratum. Each of these parameters can be selected for updating or not. To assess the influences of using updated or non-updated parameters, we tested and compared the CV and NELUCI values of parameters (2) and (3) in both non-updated and updated conditions (Table 4). Parameter (1) was not tested, as the total number of grids did not change in the study area. It can be seen that the lowest errors occurred when both of the parameters were updated, which requires area frame re-sampling (Fig. 1C). The highest NELUCI occurred when the grids in each stratum were not re-sampled but the number of grids in each stratum was updated. There are two reasons for this. Firstly, when the old sampled grids are not re-sampled, their mean values of cotton area are greater, because the value of some sampled grids with lower values in 2011were changed to larger values in 2015. It can be seen in Table 5 that the mean values of the sampled grids that were not re-sampled were much higher than those that were. Secondly, the weight of the 5th stratum, which had the highest cotton area, increased in 2015, while the weight of the 1st stratum, having the lowest cotton area, decreased. For these two reasons, the estimated total cotton area is much higher than the actual area. The highest CV values arose when both of these parameters were not updated as expected, because these two parameters were not updated to reflect the actual situation. It also can be seen in Table 4 that re-sampling of the sampled grids has a more significant influence on the CV and NELUCI values than updating the number of grids in each stratum. This is because according to Eq. (1), the mean values of the sampled grids in each stratum are multiplied by the number of grids in each stratum. Hence, if there are errors in the mean values of the sampled grids in each stratum, these will be multiplied by the number of grids. Meanwhile, both the CV and NELUCI values are greater than 5% when these two parameters are not updated; which is the exact situation that arises in the traditional method (Fig. 1A).

**Table 4:** Influences of using updated or non-updated parameters on the NELUCI and CV.

**No. of area frames in each stratum**	**Re-sampling of sampled area frames**	**NELUCI**	**CV**
Not updated	Not updated	0.1373	0.0711
Not updated	Updated	−0.0520	0.0250
Updated	Not updated	0.2147	0.0590
Updated	Updated	0.0379	0.0237

**Table 5:** Influences of using updated or non-updated parameters on strata weights and mean values of the sampled grids.

**Stratum no.**	**2011**		**2015**	
	**Stratum weight**	**Mean value of sampled area frames without resampling**	**Stratum weight**	**Mean value of sampled area frames with resampling**
1	0.3351	0.1650	0.2908	0.0257
2	0.0898	0.4500	0.0794	0.2650
3	0.1155	0.7700	0.1172	0.5367
4	0.2153	0.7760	0.2118	0.6800
5	0.2444	0.9367	0.3008	0.9143

### Advantages and disadvantages of the proposed method

To evaluate the usability of non-updated area sampling frames, and to estimate the non-sampling errors, in this study we proposed a novel method based on remote sensing data. We also defined the non-sampling error according to NELUCI that indicates the non-sampling errors introduced by using non-updated area sampling frame. We found that the non-sampling error is affected by various parameters (i.e., total number of grids, the number of grids in each stratum, and the mean value of sampled grids in each stratum), all of which can be easily calculated from remote sensing data. Importantly, we found that if these parameters are not updated, greater errors are introduced, especially for the mean value of sampled grids in each stratum. In contrast, the traditional method supposes that land use does not change greatly over the 5–10 year period after the sampling design are done. Non-sampling errors were also considered to be very low. Land use change can only be identified when a ground survey is conducted, after which it is too late for re-sampling. Compared to the traditional method, our proposed method using remote sensing has two advantages. Firstly, non-sampling errors introduced by land use changes can be estimated using the NELUCI. Secondly, non-sampling errors can be estimated using data extracted from medium and high spatial resolution imagery before a ground survey is applied, thus reducing the costs associated with invalid ground surveys and saving time on resampling (Brink & Eva, 2009).

There are several considerations that require further investigation to assess this new method’s broad applicability and overall accuracy. Firstly, to date, this method is only proposed for use with agricultural statistics based on stratified sampling; therefore, further research is needed on other sampling methods such as multivariate probability proportional to size sampling (MPPS).

Secondly, this method is based on land use data mapped by remote sensing techniques. Thus, the accuracy of land use data will affect its performance, especially in the estimation of sampled grid values based on high spatial resolution remote sensing data (Comber et al., 2012; Congalton, 1991; Sanli & Delen, 2018). To show the influences of spatial resolution and land surface complexity, we mapped crops in Bole and Suzhou using 2 m GF-1-PMS data and 0.5 m imagery. The land surface of Bole is homogeneous, with field plots larger than 0.5 km^2^; while it is complex and heterogeneous in Suzhou, with field plots smaller than 0.01 km^2^. We found that the overall accuracy of crop maps based on GF-1-PMS data in Suzhou was only 77.54%, while the 0.5 m imagery could map crops with an accuracy greater than 90% in both Bole and Suzhou (Wu et al., 2017). Since the mapping accuracy of land cover types in sampled grids based on remote sensing data has an important impact on the sampling error, we recommend the spatial resolution to be finer than 2 m in homogeneous areas and 0.5 m in complex heterogeneous areas.

Thirdly, this method (1) estimated cotton areas by area resampling frames and (2) estimated non-sampling errors while area sampling frame was non-updated, based on remote sensing data of a small area in Bole, Xinjiang, China. More studies are needed to evaluate the performance of this method over broader areas and with a greater variety of crops. Furthermore, the performance of this method should be compared with calibration estimating methods in future work (Czaplewski & Catts, 1992; Deville & Särndal, 1992; Li et al., 2014).

Finally, the grid size used also influences the outcomes of the proposed method. First, sampling methods are based on an assumption that the grids are independent. This requires a low spatial correlation between grids. If the spatial correlation between grids is high, the results will be unreliable. For example, when the grid size is 100 m × 100 m, the Moran *I* index is 0.80. If the field plots are larger than 0.5 km^2^, a situation can arise where two sampled grids cover the same plot. This means that one of the sampled grids is invalid. Second, the size of the grid has an effect on each parameter in the sampling, e.g., the total number of grids, the number of grids in each stratum, and the mean value of sampled grids in each stratum. It also has a significant impact on sampling efficiency and cost. Thus, the influence of grid size is important and should be investigated in future work.

## Conclusions

To reduce the costs of agricultural research, complete area sampling frames are commonly used for five to ten years before being updated. However, this approach is prone to non-sampling errors if the land use changes in that time. To evaluate such non-sampling errors using non-updated area sampling frame, a novel method using remote sensing was proposed in this study. We determined an approach for calculating the non-sampling errors arising from land use changes, which were readily extracted from remote sensing data. We found that:

(1) When a non-updated area sampling frame is used with stratified sampling, the total number of grids, the number of grids in each stratum, and the mean value of the sampled grids in each stratum are affected by land use changes and, therefore, should be updated. When these parameters are updated with remote sensing data, the cropping area sizes in Bole, Xinjiang, China, were estimated with a coefficient of variation of 0.0237 and NELUCI of 0.0379. These are 0.0474 and 0.0994 lower than errors calculated by traditional methods based on a non-updated area sampling frame and selected sampling units.
(2) The mean values of the sampled grids in each stratum have a more significant influence on the CV and NELUCI values when the land use changes. When high spatial resolution remote sensing data is used to estimate the values of sampled grids based on AOIs obtained from ground survey data, we recommend the spatial resolution to be finer than 2 m in homogeneous areas and 0.5 m in complex heterogeneous areas.

##  Supplemental Information

10.7717/peerj.5824/supp-1 Supplemental Information 1 Cotton area ratio in each sampling grid in 2011

10.7717/peerj.5824/supp-2 Supplemental Information 2 Cotton area ratio in each sampling grid in 2015

## Additional Information and Declarations

### Data Availability

The following information was supplied regarding data availability: The raw measurements are available in the Supplemental Files.
